# The Ethics of Producing *In Vitro* Meat

**DOI:** 10.1111/japp.12056

**Published:** 2014-02-27

**Authors:** G Owen Schaefer, Julian Savulescu

**Affiliations:** 1 Oxford Uehiro Centre for Practical EthicsSuite 8, Littlegate House, St Ebbes Street, Oxford, OX1 1PT, UK

## Abstract

The prospect of consumable meat produced in a laboratory setting without the need to raise and slaughter animals is both realistic and exciting. Not only could such *in vitro* meat become popular due to potential cost savings, but it also avoids many of the ethical and environmental problems with traditional meat productions. However, as with any new technology, *in vitro* meat is likely to face some detractors. We examine in detail three potential objections: 1) *in vitro* meat is disrespectful, either to nature or to animals; 2) it will reduce the number of happy animals in the world; and 3) it will open the door to cannibalism. While each objection has some attraction, we ultimately find that all can be overcome. The upshot is that *in vitro* meat production is generally permissible and, especially for ethical vegetarians, worth promoting.

## 1. Introduction

On 5 August 2013 a simple pan-fried hamburger was served in London alongside lettuce, tomato and a bun to three diners in front of a gaggle of gathered journalists. The burger was not the product of a world-class chef or, by any account, a revelatory gastronomic experience. Nevertheless, history was made that day because the simple burger was not produced from the meat of a slaughtered cow, pig, or any other living animal. Instead, it was produced in a laboratory by Mark Post and his team at Maastricht University — the first public demonstration of an edible *in vitro* meat product.^1^

The traditional model of meat production involves raising non-human animals to a certain age, feeding them, housing them, and ultimately slaughtering them in order to produce steaks, fillets, cutlets and other products for our consumption. But Post's demonstration shows that we could cut out the middle-man, so to speak — produce meat without involving any actual non-human animals (for simplicity, henceforth referred to as ‘animals’). Meat could be produced *in vitro* (that is, in a laboratory environment) instead of a farm. This development is exciting, but as with any new technological developments doubts will arise. This article will examine three ethical issues raised by the possibility of wide-scale production of *in vitro* meat (henceforth, IVM): disrespect for animals, reducing the number of happy animals and the possible spectre of cannibalism. While these concerns are worth discussing, we argue that they are not adequate grounds for opposing the wide-scale production of IVM.

Interest in synthesising consumable IVM — something that is more or less identical to regular meat at a cellular level, unlike meat-substitutes made from tofu, beans, mushrooms, etc. — has boomed in recent years, and Post is one among many researching IVM's viability. In 2005, Edelman^2^ and colleagues sketched the various pathways that might be used to develop *in vitro* meat. Half a decade later, a number of researchers have succeeded in the crucial first step — synthesising muscle tissue and other animal components.^3^ Likely researchers from other laboratories employing different techniques will now attempt to follow up on Post's success with products of their own.

There are some further barriers to the development of marketable IVM. The current cost (Post's burger ran around $300,000) is extraordinary, to be sure, but over time those costs may well plummet, just as genome sequencing costs have fallen precipitously since the first sequence. Also, IVM needs to be made palatable — Post's dry and bland hamburger would not make the grade in a public inundated with a plethora of mouth-watering dining options. And, of course, IVM needs to be rigorously tested for safety before it is ready for the mass market. But once the technology is in place, prices have fallen and safety has been ensured, there is some reason to expect the wide-scale production of IVM and potential replacement of farming as the typical method of food production.

Still, the growing vegetarian, organic and local food movements point toward a large, more conscientious consumer base — won't they resist IVM? But on the contrary, ethics and the environment are among the main advantages of IVM. Ethical vegetarians (e.g. Singer, 1975; Regan, 1983; Degrazia, 1996)^4^ object to consuming meat and other animal products because of the conditions imposed on animals: they are confined, disfigured, cruelly handled and painfully slaughtered. To cite just two visceral examples, egg-laying chickens frequently have their beaks partially amputated without anaesthetic, a process that unsurprisingly induces significant suffering in both short-term and long-term.^5^ And the tight confines used to raise pigs have been shown to cause lesions, foot and leg problems and psychological damage.^6^ There is some further disagreement over whether the killing of animals (who may lack a robust concept of their own lives) is in itself wrong, but they all agree the immense amount of suffering caused by factory farming is deeply unethical.

IVM production, however, does not rely on the cruel factory conditions that are widespread today. If the standard factory farm were replaced by IVM laboratories, this would have more or less the same effect of reducing animal suffering and/or slaughter as converting everyone to vegetarianism. Ethical vegetarians, then, should be among the most enthusiastic supporters and promoters of IVM. Indeed, the animal advocacy group People for the Ethical Treatment of Animals have offered a large cash prize ($1 million) to the first group to produce marketable IVM. Moreover, ethical vegetarians who think we have an obligation to avoid eating meat in order to reduce the suffering associated with factory farming should support a *pro tanto* obligation to support IVM research and production, if IVM would lead to far fewer factory farms. And even if the product does not quite replace regular factory farms, it may help those consumers who are ‘on the fence’ — they would like to be vegetarians for ethical reasons, but just love the taste of meat too much to convert. IVM would allow such weak-willed individuals to consume tasty hamburgers and steaks without worrying about causing animal suffering.

Independent of the ethical arguments themselves, there is reason to think there is a strong market for ‘ethical’ meat products. Consider the relatively recent proliferation of ‘cage-free’ and ‘free-range’ labels as well as the popularity of meat substitutes. Indeed, one survey found that, while most Americans do not rank animal welfare concerns very highly, they nevertheless believe that farm animals should not suffer.^7^ Another consumer preference survey suggests that the greatest barrier to the expansion of the meat substitute market is not the paucity of the ethical arguments, but the ‘sensory quality and resemblance to meat’ of the substitutes.^8^ IVM could easily fill a niche in the market.

IVM may also be welcomed by environmentalists. Current animal farming methods produce a large amount of pollution and resources, in large part because of the need to raise animals from birth to slaughter.^9^ In addition, even non-animal farming methods also involve a significant amount of harm to local wildlife, both directly through ploughing and harvesting as well as indirectly through the use of various pesticides.^10^ With IVM, however, much less land and water will be needed and fewer pollutants, including greenhouse gasses, will be emitted — posing less of an overall environmental burden than factory farms.^11^ Given the controlled environment in which IVM would be produced, it would also be less likely to transmit infectious agents like Mad Cow Disease or e-coli. In addition, widespread IVM production will likely lower the demand for food like cornmeal to feed animals. This will either reduce the intensity of the farmland (and thus reduce the environmental burden of such farming) that produces the animals' food, or reduce food prices and potentially help alleviate famines in certain parts of the world.

While the above considerations suggest that IVM could compete with traditional meat in the near future, fully evaluating the prospects for widespread IVM is outside the scope of this article. Still, these factors suggest a strong *prima facie* case for why we should welcome and encourage the development of IVM.

The reduction in animal suffering is perhaps the most morally salient reason to support research into and production of IVM. However, it is important to note that not all contemporary farming practices involve inflicting significant amounts of harm on animals. There has been a recent movement towards less-intensive farming, with many marketing animal products with ‘cage-free’ and ‘free-range’ labels. Also, more traditional farming methods such as those employed by the US Amish and Mennonite communities avoid many of the modern practices such as close confinement and mutilation that induce a significant amount of suffering. Arguably, at least some of these farming practices lead to relatively ethical farms — places where the animals have what seem to be overall quite good lives (the animals are well-fed, allowed to roam and socialise, minimally interfered with and kept safe from predators). It is important to note that supporting IVM is perfectly compatible with the continued existence of such happy farms, whether through direct demand for ‘natural’ meat or potential state subsidies if necessary.

Still, any technological development will be met with detractors. While IVM is too much in its infancy to attract many full-fledged objections, the emergence of some resistance is likely inevitable. Hopkins and Dacey (2008)^12^ have surveyed a large number of such potential objections, in the hopes of both encouraging a debate on the issue as well as providing pre-emptive responses. Their survey is extensive; it includes issues such as potential dangers, the authenticity of meat, moral cowardice, the ‘wrong sorts of reasons’, moral taint and the need for humility in technological progress. While these problems are interesting, each issue is only given a brief discussion. In this article, we will focus on three salient concerns about IVM and discuss each in detail. While each has its appeal, the upshot will be that none are ultimately persuasive reasons to oppose the widespread production of IVM.

## 2. Respect

One way in which *in vitro* meat could be problematic is that it is inherently disrespectful — either to nature, or to animals themselves. We will discuss each possibility in turn.

### Respect for Nature

The idea that something could be disrespectful towards nature or problematically unnatural is rather vague and covers a wide range of concerns. To clarify the relevant concern, we can draw on Helena Siipi's taxonomy of naturalness (Siipi, 2008).^13^ Siipi delineates three broad categories: historical naturalness (concerning how something came to be), property naturalness (concerning something's present properties) and relational naturalness (concerning the relationship between people and some being or object). Insofar as IVM can provide a rough cellular facsimile of real meat, property naturalness will not be relevant. And historical objections are not very plausible — the use of innumerable inanimate tools with unnatural origins is unobjectionable, and there are no obvious reasons why unnatural origin in itself should be a special problem for IVM. Serious objections could be raised, however, concerning what IVM does to the natural relationship between people and meat (or meat-producing animals).

In this vein, Roger Scruton has argued, in defence of a certain form of meat-eating, that there is value in our relationship with the natural world. This value is meant to be a sort of secular piety, characterised by a recognition of our ‘underlying fragility and dependence, and the attitude of respect toward the world and the creatures that live in it …’.^14^ The importance of respect for natural relationships is also emphasised in various religious traditions such as Confucianism (Fan, 2005),^15^ Hinduism (Dwivendi, 1990)^16^ and Native American traditions (Dussias, 1998).^17^ Meat-eating is, on this view, permissible insofar as people recognise the consumption of meat as part of a greater pattern of interdependence and interconnectivity with the broader natural world, involving an appropriate balance between technological manipulation and unintrusiveness with the natural world.^18^ Factory farming violates this value insofar as it does not recognise any interdependence with the natural, but instead involves dominating nature and bringing it into line with human needs.

A similar criticism could be levelled against IVM: it is a subversion of our previous relationship with the natural world. It involves substituting interdependence with total independence; no longer do we need to rely on nature for our survival, but can synthesise all our necessary nutrients independently. Similar objections have been raised to synthetic biology.^19^ IVM disrespects nature insofar as it treats the natural world as simply a tool for our use, rather than a partner in a certain sort of relationship. In addition, IVM can be seen to distance ourselves from the natural world, alienating us from our origins and the creatures around us.^20^

This argument relies on a notion of respect according to which relationships of dependence are important and valuable. However, it is doubtful that dependence is intrinsically worth preserving. Suppose one had been dependent on a close friend for housing for many years. The fact that one had been dependent on that friend in the past is no reason to refuse to move out when a better living situation becomes available. One should be grateful to the friend, to be sure, but gratefulness is perfectly compatible with moving on. It is certainly true that IVM will shift the relationship people have with animals and the natural world, but change is not necessarily a bad thing. The agricultural revolution involved a massive change from a relationship of dependence on the vagaries of local ecosystems to radically controlling the environment in which plants and animals were raised. IVM can be seen in this light — moving on from our previous mode of meat-production.

Another analogy is the modern pharmaceutical industry. Through most of history, humans were dependent on natural roots, extracts and other products for medicinal effect. Modern medicine has extracted the active ingredients and produces these by mass, artificial production in the form of pills, liquids, etc. There is nothing objectionable about the loss of dependence on natural medicines in favour of modern Western medicine. Food is like medicine — it is something we need. If it can be produced with distinct ethical advantages, though artificially, it should be. There are many good objections to the modern pharmaceutical industry but few (though some) find its unnaturalness a major objection.

Even if nature is owed a certain amount of respect, respect for nature and our natural relationships with the world could be preserved through various forms of ethical farming mentioned above. The risk of totally alienating ourselves from the more ‘natural’ past could be staved off by maintaining a sufficient number of such farms. Indeed, participants at a recent *in vitro* meat workshop in the Netherlands proposed a novel compromise between the value of natural means of meat production and the benefits of IVM: raise a handful of animals, perhaps in people's own backyards, under ethical conditions while periodically retrieving tissue samples that could be used as ‘donor cells’ to generate various IVM products.^21^ This scheme could preserve the purportedly important relationships between humans and the natural world while allowing large-scale production of meat, all without many of the significant ethical, economic and environmental costs associated with traditional meat production.

### Respect for Animals' Wholeness

Many object to consuming meat because the production of meat wrongs animals; similarly, some might object that the production of *in vitro* meat wrongs animals. This analogous case against *in vitro* meat is not entirely straightforward, however. Meat production is typically taken to wrong animals because animals are (grossly) harmed in the process, through their treatment and/or slaughter. *In vitro* meat is unique, though, in that no animals need be harmed at all. Nevertheless, some scholars have pointed to ways in which animals can be wronged without being harmed. It might be said that, even though no individual animals are harmed in the process of IVM, they are in fact disrespected.

One way to characterise this disrespect is a modified version of Scruton's argument: instead of thinking about disrespect for nature, perhaps we disrespect animals themselves. One prominent thought in this vein is the objection to any interventions that diminish animals' species-typical capacities. Bernice Bovenkerk, Frans Brom and Babs van den Bergh (2002)^22^ have written, in objection to the production of eggs by chickens genetically engineered to have no brains (and so not suffer), that such would violate animals' integrity:

An animal's integrity is violated when through human intervention it is no longer whole or intact, if its species-specific balance is changed, or if it no longer has the capacity to sustain itself in an environment suitable to its species. However, when the intervention is directed toward the animal's own good, we do not speak of a violation of its integrity.^23^

The senseless chicken's integrity is violated insofar as it is made unwhole, unbalanced and non-intact. It is, in effect, a gross mutilation and depravation of the chicken's nature. This principle is more or less an attempt to systematise our intuitive reaction to the generation of such senseless lobs of flesh. Similar principles that militate against the inhibition of animals' capacities have been proposed by a number of thinkers (e.g. Balzer, Rippe & Schraber, 2000; Fiester, 2008; and Thompson, 2008).^24^ And, this framework could potentially be applied to IVM — IVM, after all, involves making meat cells that certainly lack most of animals' typical capacities and are in no way part of a whole, intact animal capable of sustaining itself.

These anti-inhibition principles, though, are not easily applicable in either the case Bovenkerk, Brom and van den Bergh propose, or IVM. In both cases, a primary reason to fundamentally alter the nature of the animal is for the animal's own good. Suffering is reduced, and no conscious animal need be slaughtered for our dietary desires. (This welfarist escape clause, where inhibition of capacities is acceptable when it is for the animals' sake, also appears in Fiester, 2008.) This does not necessarily mean that the animals in question are necessarily better off dead; instead, the intervention is a way to ensure we do not wantonly harm animals.

Moreover, the justification of the principle is in the first place mistaken. The principle is grounded more or less in our revulsion at thinking of a certain sort of egg-laying chicken. However, that revulsion is better explained as a reaction to a physically disgusting phenomenon (like a piece of rotting meat), rather than a moral violation. The only other reason to accept the principle would be an argument about mutilation; it does indeed seem cruel to (even painlessly) take a living animal and deliberately make it unwhole or unable to sustain itself. In such a light, an anti-inhibition principle only makes sense as characterising a harm to already-existing animals. But as Paul Thompson (2008) notes, in the case of IVM, there is no animal being mutilated and so the argument will not be applicable. It is cells and tissue that are created, which are more akin to a plant than any animal organism, even a non-sentient one.

One could argue that, while no individual animal is being mutilated in the process of IVM, there is a species harm — a sense in which the species is being wrongfully modified. This may give some further grounds to objections to the senseless chicken case, but not IVM. IVM does not lead to the generation of a new type of animal, but rather replaces the need for the use of animals at all in meat production. A group of cells in a petri dish, after all, do not constitute a living animal; the species of chicken, cow, pig, etc. are unperturbed by the development of IVM.

### Respect for Animals' Rights

A complication arises from certain methods employed in the development of his IVM hamburger. Some techniques, including those used by Mark Post, require the acquisition of a small amount of ‘donor’ stem cells from the relevant animal, which will then be multiplied (potentially in massive quantities) and used as the basis of IVM. The cells are acquired via a nonlethal, allegedly painless biopsy procedure.^25^ This is a far cry from the worry of Bovenkerk, Brom and van den Bergh that animals will be made unwhole, but may run afoul of another proposal that animals have inviolable rights just like humans. This view has been famously pressed by Regan (2004),^26^ and more recently by Sue Donaldson and Will Kymlicka (2011).^27^

These inviolable animal rights may be enumerated in a number of ways, but are encapsulated by the (neo-)Kantian notion that we should not treat beings (including animals) as mere means to our ends. In particular, this rules out the use of animals in nonconsensual experimentation, even if the expected benefit is quite large (just as we cannot justify using children in nonconsensual experimentation even when the benefits are quite large). And the taking of a biopsy to generate IVM is arguably just such an instance — taking animal tissue samples nonconsensually for the larger benefit of both animals and humans.

The inviolable rights perspective does not, however, categorically rule out the procurement of stem cells for IVM production, as long as it is done properly. As Donaldson and Kymlicka write, ‘Using others is legitimate if the terms of the relationship reflect and uphold the membership status of both parties rather than permanently subordinating one to the other, and this, in turn, requires (as far as possible) respecting their agency and choices.’^28^ This may be accomplished in a number of ways. Firstly, efforts should be made to ensure the biopsy obtaining ‘donor’ stem cells is indeed safe, painless and leaves minimal scarring. Secondly, perhaps donations could be obtained from the carcasses of dead animals so no pain is involved. Thirdly, one may ensure that the animal ‘donor’ lives in a free and open environment in line with Donaldson and Kymlicka's vision, not the cruel confines of factory. And fourthly, the animal ‘donor’ could be appropriately compensated for its contribution to IVM, rewarded with food, toys or other resources favoured by the species.

This does not fully overcome the problem that the animals cannot possibly consent to the ‘donation’ because they do not appreciate either the costs or benefits involved. But lack of ability to consent does not always preclude human subjects research either. Proper procedures would be parallel. Non-therapeutic research on children is permissible provided the risks are minimal and parents or legally appointed decision maker consents.^29^ In the case of animals, a responsible and respectful caretaker or other representative would need to consent and risks would have to be minimal, minimised and reasonable.

Post's acquisition of stem cells for the hamburger did not follow this procedure, to be sure; the cells came from an animal already destined for the slaughterhouse. Donaldson and Kymlicka might then object that, while IVM is in theory acceptable, the way it is currently produced by the likes of Post is objectionable. This is fair enough, but speaks more to the faults of our society as a whole (in failing to adopt their vision of ‘zoopolis’ where animals are given similar citizenship rights as humans) than the particular problems of IVM. What's more, imperfect as it is, even obtaining cells for IVM from animals already in factory farms does a great deal to significantly reduce the massive violations of rights of other animals. One ‘donation’ of stem cells could be used as the basis for a massive amount of IVM products. Taking a small amount of muscle tissue involuntarily from animals doomed to the slaughterhouse anyway seems like a small price to pay for the immense ethical benefit of a great number of animals spared a similar fate. More importantly, once techniques are viable, it would be possible to push for more and more ethical methods of obtaining donor cells — potentially without the need for a donor at all, but with wholly synthetic origin cells instead.

## 3. Happy Animals

A worry sometimes raised against ethical veganism and vegetarianism is that, for all the suffering animals undergo, their lives are nevertheless worthwhile. Roger Scruton frames this issue in the following way: ‘[T]he sacrifice would not exist, but for the sacrifice. A great number of animals owe their lives to our intention to eat them’.^30^ Similarly, F. Bailey Norwood and Jason L. Lusk write, ‘There can be no real animal liberation, as liberation is tantamount to virtual extinction. Would millions of hogs, chickens and cattle prefer not to exist than to live in a world where they are raised for human consumption and profit?’.^31^ The idea is that being farmed is actually in animals' interests, as a life in a factory farm is better than no life at all. The same thing can be said about IVM — insofar as it replaces regular animal farming, it will lead to fewer animals that overall have lives worth living, which is arguably a bad outcome.

Several problems immediately arise from this objection. It is, in the first place, not entirely clear that animals involved in intensive farming have lives worth living. The life of such animals may not consist of constant torture, but the degree of maltreatment certainly makes it plausible that such animals may be better off dead. Also, there are metaphysical problems with claiming some being is harmed or wronged by not being brought into existence. After all, in such a case there is no being in existence to be the subject of wrong or harm. So, it is difficult to argue that it is better for the (prospective) animals that we bring them into existence through farming and breeding practices. In addition, as Hopkins and Dacey (2008)^32^ point out, the objection only has force if it is always good to bring more beings with minimally-worthwhile lives into existence. This implies a view of population ethics on which one should bring into existence as many beings with minimally-worthwhile lives as is sustainable, at the expense of a smaller number of lives that are, on average, much happier. Such a state of affairs seems deeply objectionable — Derek Parfit (1984)^33^ has referred to it as a ‘repugnant conclusion’ — and is a powerful, if perhaps not decisive, reason to reject the argument in question.

However, the objection becomes more plausible when we focus less on animals produced in factory farms and look instead at ‘ethically’ farmed animals. Some proponents of ethical veganism and vegetarianism, such as Regan and DeGrazia, will still object to the final step of slaughter or the way animals are exploited along the way,^34^ but others such as Singer tend to find rearing animals (or at least those lacking in self-awareness) in such a way less unobjectionable, to the extent the animals' lives are generally pleasant and the killing of one incentivises bringing more happy animals into existence.^35^ We might go a bit further and say, for *this* class of animal, it is a good thing that they exist (avoiding the more problematic claim that it is good *for them* that they exist, we can still say there is some impersonal value to their existence). In light of this impersonal value, there might be good reason to avoid endorsing a policy that would eliminate all instances of such ‘ethical’ farming.

Would the promotion of IVM be such a policy? Will IVM replace all or even almost all instances of ‘ethical’ farming? Based on what we know, the answer seems to be no. Even if IVM becomes wildly popular, there will always be niche markets. Some consumers will prefer authentic, ‘real’ meat, raised in a traditional manner. Also, the commitments of some producers to traditional, non-intensive farming methods, such as the American Amish and Mennonite communities, will likely persevere even in the face of the great popularity of IVM. It may be that the number of ‘ethical’ farms goes down, as cage-free and free-range products currently marketed to ethically-conscious consumers are replaced by IVM, but those farms are unlikely to disappear entirely. Moreover, any impersonal value lost by the reduction in the number of ethical farms will be outweighed by the more-significant value of reducing the number of animals subject to the intense conditions of factory farming.

But this response may be unsatisfactory to those who envision a world where meat-eating is widespread and performed under humane conditions where animals have rich, pleasurable and fulfilling lives. This world could be seen as superior to one with widespread IVM consumption insofar as the ethical meat-eating world has many more happy animals than the IVM-eating world. Producing IVM is unethical to the extent that it crowds out the ‘ethical’ meat market and makes achieving that happy animal world more difficult.

There is some appeal to this picture of a world full of happy, contented animals sustained by eager but ethically conscientious meat consumers, but it nevertheless has some troubling implications. The view still implies a controversial ‘total’ view of wellbeing that aims at maximising the amount of happiness in the world in part by bringing more and more creatures into existence.^36^ So individuals would have obligations to have as many children as possible, until an additional child would not have a happy life — imposing severe and possibly unreasonable reproductive burdens on people. It also implies, oddly enough, that vegetarianism is ethically wrong because people should be buying ‘ethical’ meat, bringing more happy animals into existence. Indeed, even current consumers of ‘ethical’ meat err insofar as they do not maximise the ratio of ‘ethical’ meat to non-meat products in their diet.

Additionally, even if we accept that there is positive reason to bring about widespread rearing of happy animals, IVM is eminently defensible so long as it is substantially cheaper than the production of ethical meat. As Singer notes, the happy animals argument requires the contingent fact ‘that for economic reasons we could not rear the [animals] unless we eat them’.^37^ Yet sufficiently cheap IVM will allow for the rearing of happy animals without slaughter, while also preserving people's ability to eat actual meat. Consider that modern meat prices are as low as they are because factory farming gains efficiencies often at the expense of animals' wellbeing. Small cages, crippling weight, psychologically degrading environments and other ills exist because they make the process more efficient. Current prices of marginally more ethical meat (such as that under the ‘free-range’ label) are higher because it is simply more costly to raise, house, maintain and slaughter animals ethically. And to truly meet the standards of the happy animal arguments, conditions will have to be better (and so prices higher) than many current allegedly ethical meat products. Conversely, there is also some reason to think that IVM could eventually be produced for sale at prices similar to that of current factory farm meat. IVM does not require resource-intensive feeding, confinement, rearing and slaughter of animals, potentially cutting costs even more than factory farming has.

For this reason, it is possible that if low-cost IVM were achievable (which is necessary for it to become widespread anyway), then we may have a morally superior option to the consumption of ethical meat: consume IVM, and reserve the cost savings from choosing IVM over ethical meat to pay for nature preserves where animals could live long, happy and fulfilling lives. (Indeed, a similar and presently feasible vegetarian-plus-nature-preserve strategy is preferable to the purchase of expensive ethical meat.) Maintaining the preserves should be cheaper than raising ethical meat, as there is no need for well-located farms, slaughterhouses, fattening diets and other resources associated with meat production. In any case, the animals would be better off on nature preserves than on even ethical farms insofar as they live longer and freer in their natural habitats than they could under a feasible ethical meat production program. Consumers may be unwilling to actually donate their savings in this way, but again that speaks against the motivation of consumers, not the ethicality of IVM.

Finally, there are strong environmental arguments to limit the number of happy animals (and, for that matter, humans). Maintaining livestock requires land not just for the animals themselves, but additionally for the food they eat — a double cost not present with IVM. And traditional meat production involves a large amount of pollution from the animals themselves, including greenhouse gasses.^38^ Large-scale maintenance of even happy animals for slaughter may then pose environmental hazards. Some moderation in even happy animal farming will be required. This balancing of environmental concerns with demands for meat would leave considerable space for a market in IVM in addition to a market of happy animals. Most importantly, IVM and ethical meat need not be exclusive. Both could coexist and together exert maximum social pressure against unethical meat production.

## 4. Cannibalism

The ability to synthesise meat may not only change the way meat is produced, but also expand the range of meat that can be produced. This may include endangered species, difficult-to-domesticate animals, extinct animals — and, perhaps most disturbingly, humans. Cannibalism is indeed a near-universal taboo in contemporary society, and IVM raises the spectre of a new, easily-produced human flesh for the consumption of a small minority who might want to engage in or experiment with cannibalism. Though cannibalism is unlikely to become a widespread craze, there will be at least some people with strange predilections, unusual cultural norms or perhaps just curiosity about the taste of human flesh^39^ who might be interested in developing and consuming human IVM. This could be viewed as a risk of IVM, opening the door to the facilitation of a depraved practice.^40^ It is a slippery slope objection to IVM.

The most obvious reaction to this possibility of human IVM is to ban it. Just as, for instance, cloning is banned in the 13 US states and the European Union for moral reasons, we could put in place strict restrictions on the synthesis of human flesh for the purpose of consumption. Given common revulsion at the prospect of cannibalism, this reaction is indeed rather likely. However, it is too quick — we should ask first, what is so wrong with cannibalism of artificially created human cells and tissue that it must be banned? This will not only help us understand whether potential cannibalism is a reason to oppose IVM, but also clarify what, exactly, is wrong with cannibalism in the first place — a topic that has received relatively little attention in the philosophical literature.

### The Harm of Cannibalism

Most actual cases of cannibalism are morally objectionable because they involve two features in addition to the consumption of human flesh: the killing of a human being, and the desecration of a corpse. We rightfully react in horror when someone is murdered for the sake of a very specific sort of meal — the taking of another person's life is one of the gravest harms possible. And, quite plausibly, consuming the victim's corpse makes the killer's action even more objectionable — it is a form of desecration. This can be construed as a sort of posthumous harm (incredible disrespect to the dead), or (if that is too controversial) in the very least a wrong to the family of the victim. In this sense, the cultural taboo against cannibalism is generally justified on grounds other than the repulsiveness of the act.

But IVM importantly involves neither of those factors. No human would be killed in the process of producing human IVM, nor would any corpse be desecrated. So is cannibalism *per se* wrong? Prosecution for cannibalism itself is very rare. The most famous convictions of cannibals, such as the 19^th^-century crew adrift at sea^41^ or more recently Armin Meiwes,^42^ were not for cannibalism itself but murder and (in the case of Meiwes) desecration of a corpse. This is simply because cannibalism is rarely outlawed. Idaho is the only US state to explicitly ban it, and few other jurisdictions internationally have similar statutes.^43^ Indeed, there is not much need to ban cannibalism — murder charges are sufficient to exact justice on those who would kill in order to taste human flesh.

We might never want to partake in such cannibalism ourselves, but a distaste for something is no reason to think it immoral — just as a distaste towards eggplant is no reason to think its consumption immoral. Interestingly, whether there is any further wrong to cannibalism — over and above the killing and desecration — has received scant attention in the literature, perhaps because it is rarely of practical relevance nowadays. The discussions that do exist are quite brief, but offer some sense of how an argument for the further wrongness of cannibalism might go. We will now consider two such arguments.

### Respect for People

Frederick Ferré (1986) has argued that cannibalism is wrong because it is disrespectful to the inherent value of humans:

Psychological grounds aside, the strongest ethical ground for the avoidance of eating human flesh is the familiar principle of due respect for inherent value. Human beings are entities so complex as to be capable of the most creative and free mental activities known in the universe. It would be gross disrespect for such qualitative excellence — the capacity for intense consciousness of being for oneself — to look at such an entity and see only meat.^44^

Ferré does go on to note the further problem that cannibalism threatens people's lives, but he appears to take disrespect to be a wrong independent of the loss of life. This demand for respect is grounded in humans' unique psychological capacities; consuming human flesh is taken to be a denial of the significance of such capacities, and cannibals instead see humans as an object to be consumed.^45^

Importantly, such a view would account for many people's intuitions that cannibalism is wrong even if fully voluntary, such as in the case of Armin Meiwes. In Germany in 2001, Bernd Jurgen Brandes consented to be killed and eaten by Meiwes (at least according to Meiwes). This case caused international outrage and Meiwes was found guilty of murder and disturbing the peace of the dead.^46^ Such a case illustrates the intuition that there are some harms which one should not be legally allowed to consent to, even if fully competent. The wrongness of such an act might lie in part in the failure to respect the person, our fundamental humanity. For this reason, J. S. Mill objected to selling oneself into slavery.^47^

This might well be a solid grounding for the wrongfulness of typical cases of cannibalism over and above killing and desecrating a corpse. There, Ferré would say, the cannibal is disrespectful towards his or her victim, even if the victim is oneself. The argument might seem to have problematic implications — does it mean we could not synthesise human tissue for the sake of medical procedures like blood transfusions or organ transplants, due to the possibility of seeing other humans just as medical resources? Ferré, though, could point to a life-saving exemption to the prohibition. Just as we forgive cannibals who only eat the flesh of humans for the sake of survival, such as the survivors of the 1972 Andes flight disaster,^48^ life-saving organ synthesis could be seen as permissible due to the extremity of the benefit.

There is, however, a further reason to doubt that Ferré's principle would be applicable in the present case: with human IVM, there is no human being involved in the process who could be disrespected. No human being is being used as meat instead of as a being deserving of equal consideration, and no human is deprived of the capacities Ferré claims are central. It is mere human tissue and cells that are used for food. There is no ‘failure to respect human persons’ objection to giving one's cells and tissues to be used for medical purposes, or to using those of another, or even those created by stem cell technology.

It might be said that there is disrespect towards humans as a species in producing human IVM. However, how this disrespect would manifest itself is not clear. True, human flesh would be used in a way that does not directly involve recognising humans' inherent worth. But it is not being used in a way that subverts or undermines our inherent value, just as using human cells and tissue for medical purposes expresses rather than subverts our nature as persons. Alternatively, it might be that initial human IVM production requires a human ‘donor’ to provide genetic or tissue samples that would serve as the model for future products. However, this just means that the donor must give their proper informed consent; once consent has been obtained, there would be little reason to worry the donor is being treated disrespectfully.

## 5. Conclusions

The preceding discussion has covered three potential worries about the production of IVM — violation of respect, reduction in happy animals and facilitation of cannibalism. While these worries are relevant, none of them are sufficient to ground serious opposition to the development of IVM. Other, more sound, objections are in theory possible, and it remains to be seen whether in the coming years such will appear in the literature. In the absence of such convincing objections, though, the *prima facie* case for promoting IVM — less animal slaughter and suffering, less pollution, potentially lowered costs, etc. — is powerful enough to show that we should be supportive of the continued research into and ultimate production of IVM. At the very least, IVM could coexist with ethical meat production to maximally reduce factory and other unethical farming practices. Perhaps, it will ultimately replace rearing animals for meat, but that is not necessary for our argument to support vigorous research into the development of IVM. And if ethical vegetarians are right that we have strong obligations to reduce our infliction of animal suffering and death, we may have similarly strong (though still *pro tanto*) obligations to support research into IVM in order to ensure that it becomes marketable as soon as possible.^49^

## Notes

1 Henry Fountain, ‘A lab-grown burger gets a taste test’, *The NewYork Times*, 5 August (2013). Available at: http://www.nytimes.com/2013/08/06/science/a-lab-grown-burger-gets-a-taste-test.html

2 P. D. Edelman, D. C. McFarland, V. A. Mironov & J. G. Matheny. ‘Commentary: In vitro-cultured meat production’, *Tissue Engineering*11,5–6 (2005): 659–662, doi:10.1089/ten.2005.11.659.

3 M. Langelaan, K. Boonen, R. Polak, F. Baaijens, M. Post & D. van der Schaft. ‘Meet the new meat: Tissue engineered skeletal muscle’, *Trends in Food Science &Technology*21,2 (February 2010): 59–66, doi:10.1016/ j.tifs.2009.11.001; Datar, I. &M. Betti, ‘Possibilities for an in vitro meat production system’, *Innovative Food Science & Emerging Technologies*11,1 (2010): 13–22, doi:10.1016/j.ifset.2009.10.007.

4 Peter Singer, *Animal Liberation*(New York: Avon Books, 1975); Tom Regan, *The Case for Animal Rights*(Berkeley, CA: University of California Press, 2004); David DeGrazia, *Taking Animals Seriously: Mental Life and Moral Status*(Cambridge: Cambridge University Press, 1996).

5 See J. Breward & M. J. Gentle, ‘Neuroma formation and abnormal afferent nerve discharges after partial beak amputation (beak trimming) in poultry’, *Experientia*41,9 (1985): 1132–1134, doi:10.1007/ BF01951693; Michael Gentle, Louise N. Hunter & David Waddington. ‘The onset of pain related behaviours following partial beak amputation in the chicken’, *Neuroscience Letters*128,1 (1991): 113–116, doi:10.1016/0304-3940(91)90772-L.

6 See B. E. Rollin & Paul B. Thompson, ‘Perspectives on emergence of contemporary animal agriculture in the mid-twentieth century:The decline of husbandry and the rise of the industrial model’, in W. G. Pond, F.W. Bazer, B. E. Rollin & P. B. Thompson (eds) *AnimalWelfare in Animal Agriculture: Husbandry Stewardship, and* *Sustainability in Animal Production*(Boca Raton, FL: CRC Press, 2012), pp. 3–12.

7 See F. Bailey Norwood & Jayson Lusk, *Compassion, by the Pound: The Economics of Farm AnimalWelfare*(Oxford: Oxford University Press, 2011).

8 See Annet C. Hoek, A. Pieternel Luning, Pascalle Weijzen, Wim Engels, Frans J. Kok & Cees de Graaf, ‘Replacement of meat by meat substitutes: A survey on person- and product-related factors in consumer acceptance’, *Appetite*56,3 (2011): 662–673, doi:10.1016/j.appet.2011.02.001.

9 See Henning Steinfeld, Food and Agriculture Organization of the United Nations & Livestock, Environment and Development (Firm), *Livestock's Long Shadow: Environmental Issues and Options*(Rome: Food and Agriculture Organization of the United Nations, 2006).

10 Steven Davis, ‘The least harm principle may require that humans consume a diet containing large herbivores, not a vegan diet’, *Journal of Agricultural and Environmental Ethics*16,4 (2003): 387–394; Maurice E. Pitesky, Kimberly R. Stackhouse & Frank M. Mitloehner. ‘Clearing the air’ in D. Sparks (ed.) *Advances* *in Agronomy*, no. 103 (Burlington, MA: Academic Press/ Elsevier, 2009), pp. 1–40. Available at: http:// linkinghub.elsevier.com/retrieve/pii/S0065211309030016.

11 See Hanna L. Tuomisto & M. Joost Teixeira de Mattos, ‘Environmental impacts of cultured meat production’, *Environmental Science & Technology*45,14 (2011): 6117–6123, doi:10.1021/es200130u.

12 Patrick D. Hopkins & Austin Dacey, ‘Vegetarian meat: Could technology save animals and satisfy meat eaters?’ *Journal of Agricultural and Environmental Ethics*21,6 (2008): 579–596, doi:10.1007/s10806-008- 9110-0.

14 Helena Siipi, ‘Dimensions of naturalness’, *Ethics & the Environment*13,1 (2008): 71–103.

14 Roger Scruton, ‘The conscientious carnivore’, in S. F. Sapontzis (ed.) *Food for Thought: The Debate Over Eating Meat*(Amherst, NY: Prometheus Books, 2004), pp. 81–91.

15 Ruiping Fan, ‘A reconstructionist Confucian account of environmentalism: Toward a human sagely dominion over nature’, *Journal of Chinese Philosophy*32,1 (2005): 105–122, doi:10.1111/j.1540- 6253.2005.00178.x.

16 O P. Dwivendi, ‘Satyagraha for conservation:Awakening the spirit of Hinduism’, in J. R. Engel & J. G. Engel (eds) *Ethics of Environment and Development?: Global Challenge, International Response*(London: Belhaven Press, 1990).

17 Allison M. Dussias, ‘Asserting a traditional environmental ethic: Recent developments in environmental regulation involving Native American tribes’, *New England Law Review*33,3 (1999): 653– 666.

18 See Donald B. Thompson, ‘Natural food and the pastoral: A sentimental notion?’ *Journal of Agricultural and Environmental Ethics*24,2 (2011): 165–194, doi:10.1007/s10806-010-9245-7.

19 See M. K. Cho, David Magnus, Arthur L. Caplan, Daniel McGee & the Ethics of Genomics Group, ‘Ethical considerations in synthesizing a minimal genome’, *Science*286,5447 (1999): 2087–2090, doi:10.1126/ science.286.5447.2087.

20 See Simon Fairlie, *Meat?: A Benign Extravagance*(East Meon: Permanent Publications and White River Junction, VT: Chelsea Green Publishing Co., 2010).

21 Cor van der Weele, ‘In vitro meat, ethics and design’. Paper presented at the Fourth Annual Dutch Conference on Practical Philosophy, Eindhoven, the Netherlands, 2 November 2012.

22 Bernice Bovenkerk, Frans W. A. Brom & Babs J. van den Bergh, ‘Brave new birds: The use of “animal integrity” in animal ethics’, *The Hastings Center Report*32,1 (2002): 16, doi:10.2307/3528292.

23 Ibid., p. 21.

24 Philipp Balzer, Klaus Peter Rippe & Peter Schaber, ‘Two concepts of dignity for humans and non-human organisms in the context of genetic engineering’, *Journal of Agricultural and Environmental Ethics*13 (2000): 7–27; Autumn Fiester, ‘Justifying a presumption of restraint in animal biotechnology research’, *The American* *Journal of Bioethics*8,6 (2008): 36–44, doi:10.1080/15265160802248138; Paul B. Thompson, ‘The opposite of human enhancement: Nanotechnology and the blind chicken problem’, *NanoEthics*2,3 (2008): 305–316, doi:10.1007/s11569-008-0052-9.

25 Maria Cheng, ‘Q &A on the science of growing hamburger in a lab’, *Yahoo News*(via the AP) (2010): 5 August. Available at: http://news.yahoo.com/q-science-growing-hamburger-lab-115332943.html

26 Regan op. cit.

27 Sue Donaldson & Will Kymlicka, *Zoopolis*(Oxford: Oxford University Press, 2011).

28 Ibid., p. 135

29 Department of Health, *Reference Guide to Consent for Examination orTreatment*, 2nd edn. (London: Gov.UK, 2009). Available at: https://www.gov.uk/government/uploads/system/uploads/attachment_data/file/138296/ dh_103653__1_.pdf

30 Scruton op. cit.

31 Norwood & Lusk op. cit., p. 186.

32 Hopkins & Dacey op. cit.

33 Derek Parfit, *Reasons and Persons*(Oxford: Clarendon Press, 1984). Available at: http://public.eblib.com/EBLPublic/PublicView.do?ptiID=728732

34 See also Richard P. Haynes, ‘The myth of happy meat’, in D. M. Kaplan (ed.) *The Philosophy of Food*(Berkeley, CA: University of California Press, 2012), pp. 161–168; Gary L. Francione, ‘Animal welfare, happy meat and veganism as the moral baseline’, in D. M. Kaplan (ed.) *The Philosophy of Food*(Berkeley, CA:University of California Press, 2012), pp. 169–189.

35 Peter Singer, ‘Killing humans and killing animals’, *Inquiry*22,1–4 (1979): 145–156, doi:10.1080/ 00201747908601869; Peter Singer, *Practical Ethics*(Cambridge: Cambridge University Press, 2011).

36 Singer, 2011 op. cit.

37 Singer, 2011 op. cit. p. 120.

38 Steinfeld *et al*. op. cit.

39 See, e.g., William Buehler Seabrook, *JungleWays*(London: George C. Harrap and Company, 1931).

40 Jim Thomas, ‘Flask-grown flesh’, *The Ecologist*20 July (2008). Available at: http://www.theecologist.org/ blogs_and_comments/commentators/Jim_Thomas/269449/flaskgrown_flesh.html; Hopkins and Dacey op. cit.; Fairlie op. cit..

41 *Regina v. Dudley and Stephens*14 Q.B.D. 273 (1884). Available at: http://cyber.law.harvard.edu/eon/ei/elabs/majesty/stephens.html

42 Associated Press, ‘German court sentences cannibal to life in jail’, *NBC News*(2006): 9 May. Available at: http://www.nbcnews.com/id/11909486/

43 See Carmen M. Cusack, ‘Placentophagy and embryophagy: An analysis of social deviance within gender, families, or the home’, *Journal of Law and Social Deviance*1 (2011): 112–168.

44 Frederick Ferré, ‘Moderation, morals and meat’, *Inquiry*29,1–4 (1986): 391–406, at p. 403, doi:10.1080/ 00201748608602094.

45 For a similar argument, see Cora Diamond, ‘Eating meat and eating people’, in C. Sunstein and M. Nussbaum (eds) *Animal Rights*(Oxford: Oxford University Press, 2011), pp. 94–106. Available at: http:// www.oxfordscholarship.com/view/10.1093/acprof:oso/9780195305104.001.0001/acprof-9780195305104

46 Associated Press op. cit.

47 John Stuart Mill, *On Liberty*, ed. E Alexander (Peterborough, ONT: Broadview Press, 1999).

48 See Hans Askenasy, *Cannibalism: From Sacrifice to Survival*(Amherst, NY: Prometheus Books, 1994).

49 This work was subsidised by the generous support of theWellcomeTrust, grant numberWT086041/Z/08/Z.

